# Laser Doppler Blood Flow Imaging Using a CMOS Imaging Sensor with On-Chip Signal Processing

**DOI:** 10.3390/s130912632

**Published:** 2013-09-18

**Authors:** Diwei He, Hoang C. Nguyen, Barrie R. Hayes-Gill, Yiqun Zhu, John A. Crowe, Cally Gill, Geraldine F. Clough, Stephen P. Morgan

**Affiliations:** 1 Electrical Systems and Optics Research Division, Faculty of Engineering, University of Nottingham, University Park, Nottingham NG7 2RD, UK; E-Mails: diwei.he@nottingham.ac.uk (D.H.); hoangnc2k@yahoo.com (H.C.N.); barrie.hayes-gill@nottingham.ac.uk (B.R.H.-G.); yiqun.zhu@nottingham.ac.uk (Y.Z.); john.crowe@nottingham.ac.uk (J.A.C.); 2 School of Medicine, University of Southampton, University Road, Southampton SO17 1BJ, UK; E-Mails: cag1@soton.ac.uk (C.G.); G.F.Clough@soton.ac.uk (G.F.C.)

**Keywords:** laser Doppler, blood flow, CMOS, imaging sensor, full field

## Abstract

The first fully integrated 2D CMOS imaging sensor with on-chip signal processing for applications in laser Doppler blood flow (LDBF) imaging has been designed and tested. To obtain a space efficient design over 64 × 64 pixels means that standard processing electronics used off-chip cannot be implemented. Therefore the analog signal processing at each pixel is a tailored design for LDBF signals with balanced optimization for signal-to-noise ratio and silicon area. This custom made sensor offers key advantages over conventional sensors, *viz.* the analog signal processing at the pixel level carries out signal normalization; the AC amplification in combination with an anti-aliasing filter allows analog-to-digital conversion with a low number of bits; low resource implementation of the digital processor enables on-chip processing and the data bottleneck that exists between the detector and processing electronics has been overcome. The sensor demonstrates good agreement with simulation at each design stage. The measured optical performance of the sensor is demonstrated using modulated light signals and *in vivo* blood flow experiments. Images showing blood flow changes with arterial occlusion and an inflammatory response to a histamine skin-prick demonstrate that the sensor array is capable of detecting blood flow signals from tissue.

## 1. Introduction

Laser Doppler blood flow measurements [1–3] have been used as a clinical tool for measuring microcirculation in superficial tissue for many years. Its wide range of clinical applications includes studies of allergic reactions [4], burn depth assessment [5], skin cancer diagnosis [6], assessment of skin diseases [7] and investigating the effects of transdermal drug delivery [8]. Blood flow is a velocity dependent parameter which has been defined as

(1) $$\text{Flow}=\int_{\omega_{1}}^{\omega_{2}}\omega P(\omega )d\omega$$

where ω_i_ = 2πf_i_, i = 1,2, f_1_ and f_2_ are typically 20 Hz and 20 kHz respectively [2]. P(ω) is the power spectrum of the photocurrent fluctuations. The integral is often normalized by the power of the DC signal to remove the effects of laser power fluctuations and skin reflectance variations.

The first skin blood flow measurement was performed by Stern [1] over 30 years ago, using a low power collimated beam to illuminate a small area of skin. In clinical applications such as studies of burn depth assessment and plastic surgery the spatial variations in blood flow are important, tissue perfusion should therefore be assessed by an imaging technique rather than a single point method. Some solutions use a single laser beam scanning over an area of interest to build up a flow image [9,10], however, the acquisition time is relatively long due to the necessary mechanical scanning. For example a typical commercially available system [11] can take up to 5 min to obtain a 256 × 256 image (4 ms per pixel). For this reason a line scanner utilizing a 64 × 1 photodetector array has been developed which can provide 64 × 64 pixel images in 4 s [12].

An alternative technique for blood flow imaging is Laser Speckle Contrast Analysis (LASCA) [13–15], in which a full frame CCD camera is employed to acquire speckle images and a block of pixels is used to calculate the speckle contrast. According to the theory the measured speckle contrast is proportional to the velocity of the moving blood cells. Although LASCA provides a cost effective method for real-time blood flow imaging the measurement results are exposure time dependent [16] and the spatial averaging performed across a sub-array (often 5 × 5 or 7 × 7 pixels) [15] results in a reduction in spatial resolution. Furthermore a model linking measurement and flow has been demonstrated for laser Doppler blood flow (LDBF) [17] for quantitative blood flow analysis [18] whereas such models are still a subject of research at present for LASCA [16]. It has been demonstrated that multi-exposure LASCA provides more accurate quantitative flowmetry [19]. Self-mixing interferometry [20] offers the potential for full field Doppler flow imaging although to date this has been performed with low density (12 × 1) vertical cavity surface emitting laser (VCSEL) arrays [21,22] and it has been suggested that signal to noise ratio is lower than with conventional sources [23].

In recent years with the development of high frame rate CMOS technology, an implementation of full field LDBF based on a commercial CMOS image sensor coupled with a digital signal processor (DSP) has been demonstrated [24–26]. The Doppler signal detected by the sensor at each pixel is multiplexed, digitized and then transferred off chip for signal processing. The advantage of this system over the scanning laser Doppler imaging system is that the frame rate of the system is increased due to the absence of moving scanning components. However, because the flow processor requires a high number (typically 1024) of raw data frames to produce one flow image, a data bottleneck exists between the camera and the signal processing unit. This means that compromises have to be made to achieve acceptable performance. For example only 128 × 128 pixels of a 1024 × 1024 commercial CMOS array are used to form an image [25], and low resolution analog to digital converter (ADC) and lower sampling frequency [24] are used. In [25] the system requires 38 ms to capture 1024 raw frames, but takes 3096 ms to transfer the data from the camera to the PC and 998 ms for digital signal processing. The system described by Leutenegger *et al.* [26] combines a high speed commercial CMOS camera chip with a field programmable gate array and obtains Doppler blood images at 12–14 frames per second by electronically scanning a 480 × 60 pixel area. The sampling frequency at each pixel is 14.9 kHz and a 128 point fast Fourier Transform (FFT) is used to calculate flow. Technology improvements in the future will almost certainly improve to allow the 40 kHz, 1024 point FFT achieved by scanning systems to be obtained in full field. An additional drawback is that as the commercial sensors are intended for a range of applications they are not tailored for LDBF. An important consequence of this is that there is no anti-aliasing filter at the pixel level of the sensor. Serov *et al.* have noted that the relationship between velocity and frequency is non-linear [24] for frequencies above 6 kHz, which is half of the sampling frequency, due to aliasing effects. General purpose CMOS cameras also have a dynamic range problem when faced with low modulation depth LDBF signals containing a large DC background and small AC variations, especially when a low resolution ADC is used. A summary of LDBF imaging devices is shown in Table 1.

A custom made camera design offers several advantages over commercial cameras as the specifications can be tailored to the signals of interest. Such sensors based on on-chip lock-in detection have been developed for optical coherence tomography [27] and wide field sectioning microscopy [28]. For laser Doppler blood flowmetry, the pixel size, current to voltage conversion gain and number of digitization bits can be designed to best match those of typical signals. Appropriate anti-aliasing filters can also be added at the pixel level. Integration of the pixel front-end with on-chip processing enables each pixel to be sampled at a minimum rate of 40 kHz with a low data readout rate required, as the output is a processed flow image rather than a series of raw data images. In CMOS custom made designs there are considerable design constraints in terms of the silicon area of the processing electronics and the ratio between the areas of the pixel level electronics to that of the photo-detectors (fill factor). The circuits used in the discrete electronics systems cannot simply be replicated on-chip as the relatively low frequencies used in LDBF means that on-chip component sizes are commensurately often large. Therefore moving from discrete electronics at a single point to a fully integrated sensor array is a challenging task and design optimizations and compromises need to be made.

To the authors' knowledge, this paper demonstrates the design and characteristics of the first fully integrated 2D laser Doppler imaging sensor. This 64 × 64 array iteration significantly contributes to the design of a high density array Doppler imaging system. Section 2 presents the pixel design and system configuration of the sensor and Section 3 the optical set ups. Section 4 describes the experimental results of pixel front-end characterizations, a modulated light source test, a rotating diffuser and *in vivo* blood flow imaging of arterial occlusion and an inflammatory response. Discussion and conclusions follow in Section 5.

## 2. Sensor Design

**Figure 1.**
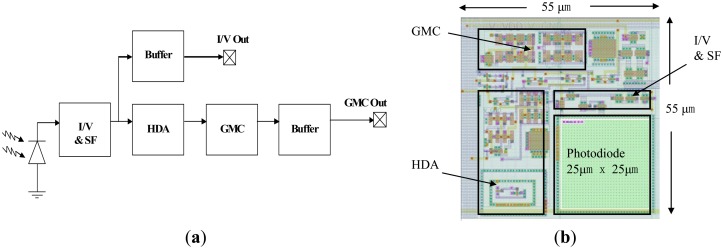
(**a**) Block diagram of the pixel; (**b**) Layout of the pixel (I/V for Current to Voltage Converter, SF for Source Follower, HDA = Hysteretic Differentiator Amplifier, GMC for Transconductance-Capacitance Low Pass Filter).

The analog signal processing at each pixel is a tailored design for LDBF signals in order to increase signal-to-noise ratio (SNR) and is optimized for silicon area. Figure 1a shows a block diagram of a single pixel on the 64 × 64 array. It consists of a photodiode, a current to voltage converter (I/V) with a source follower (SF), a hysteretic differentiator amplifier (HDA) [29], a transconductance-capacitance low pass filter (GMC) for anti-aliasing and two buffers.

Doppler shifted light is detected by the photodiode, which is of size 25 μm × 25 μm and is an n-well-p-substrate type whose responsivity is approximately 0.3 A/W at a wavelength of 667 nm [30,31]. The current to voltage converter consists of a photodiode with a load of two diode-connected P-type MOSFETs (operating in subthreshold as logarithmic wide dynamic DC range detectors) feeding into a source follower. The AC gain of the logarithmic front-end is inversely proportional to the DC photocurrent and hence the bandwidth. Therefore, as the DC photocurrent increases, the bandwidth of the circuit will also increase. At a typical DC current of ∼150 pA, the bandwidth of the current to voltage converter is ∼20 kHz. The logarithmic current to voltage converter provides a natural normalization of the AC signal by the DC light level [32] as often required in laser Doppler measurements. The output of the current to voltage converter can be observed through a buffer for more detailed sub-circuit characterization. The subsequent HDA consists of an operational transconductance amplifier (OTA) with an inverted CMOS inverter and an NMOS transistor capacitor circuit. It amplifies the AC signal by a factor of 30 [33] without DC amplification to increase the modulation depth of the signal before digitization. The GMC circuit is used as an anti-aliasing filter before the signal is digitized by the ADC, in which the cut-off frequency is set to 20 kHz as the Doppler frequency is typically in the range from 20 Hz to 20 kHz. Figure 1b shows the layout of the 55 μm × 55 μm pixel. The photodiode, I/V, SF, HDA and GMC are highlighted with individual black boxes for clarity.

**Figure 2.**
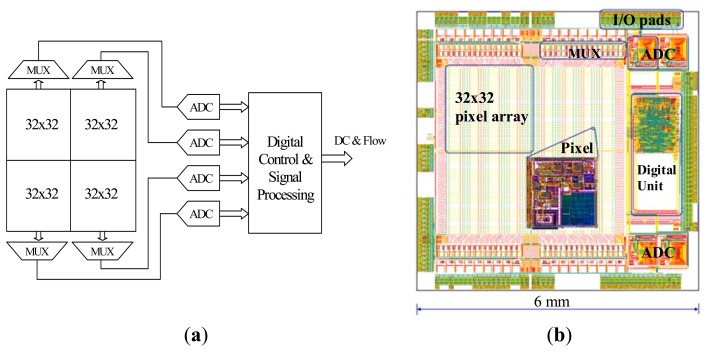
(**a**) Block diagram of the sensor (MUX for multiplexer, ADC for analog to digital converter); (**b**) Sensor layout (reproduced from [35] with permission).

Figure 2a shows a block diagram of the 64 × 64 pixel array which is divided into four identical 32 × 32 arrays, each with a dedicated multiplexer and 10-bit successive approximation register (SAR) ADC running at a sampling rate of 1.28 MHz. The pixel output voltages in each sub-array are multiplexed into the dedicated ADC. Each ADC samples a row of 32 pixels at a time and the effective sampling rate at each pixel is 40 kHz. The digitized data are then transferred to the on chip digital signal processor.

After acquiring 1024 samples for each pixel in the row, the ADC samples the next row. Therefore blood flow images are formed at ∼1 frame per second by electronically scanning each row of the four 32 × 32 pixel arrays in parallel. The digital back-end provides control signals to control the operation of the whole system and processes digitized data to produce flow parameters according to Equation (1). The digital signal processing unit employs digital filters and 512 × 32 memory bits (SRAM) to store data to be processed. The standard method of implementing the frequency weighted filter described in Equation (1) is to perform a 1024-point fast Fourier transform (FFT). However, this is not feasible on-chip due to space constraints. Infinite impulse response (IIR) filters with a low number of taps are used as low resource implementation of the data processing. Compared to the 1024-point FFT, the error in the flow calculation with IIR filters is only 0.7% [34]. The Verilog-A models of the analogue front-end and ADC were simulated in Agilent Advanced Design System (ADS) and the full-chip mixed-signal simulation was performed in Cadence AMS simulator.

The chip layout shown in Figure 2b is of size 6 mm × 6 mm and was fabricated in a 0.35 μm four metal layer CMOS process (Austria microsystems, Unterpremstaetten, Austria). Areas of the chip other than the photodiodes are covered by a metal layer to prevent illuminating parts of the chip that are not intended to be light sensitive.

## 3. Experimental Setups

This section describes four optical setups used to investigate the performance of the sensor array. Each setup progressively builds up the complexity of the detected signals. The first utilizes a modulated laser to control the illumination with different DC light level, AC amplitude and frequency to characterize the performance of the pixel analog front-end. The second configuration uses light-emitting diodes (LEDs) and an aperture to simulate predictable laser Doppler signals. The third configuration has a rotating diffuser producing more complex laser Doppler signals which are similar to real blood flow signals. Finally blood flow in a human finger with arterial occlusion and a forearm showing an inflammatory response is imaged.

### 3.1. Chip Characterization Setup

**Figure 3.**
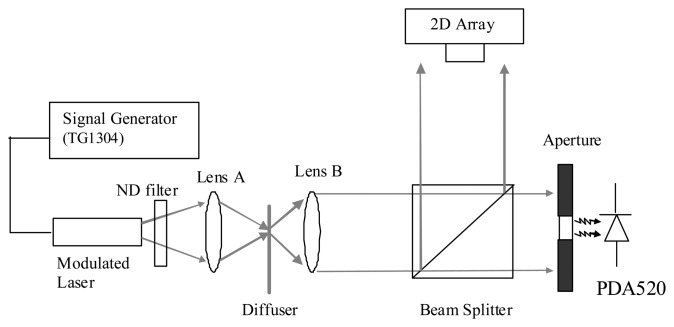
Optical setup for characterization of the pixel analog front-end (ND for Neutral Density).

The experimental setup shown in Figure 3 uses a 7 mW (635 nm) modulated laser diode (IQ1A, Power Technology, Little Rock, AR, USA) to characterize the AC and DC responses of the analog pixel front-end. The power and modulation depth of the laser are controlled by a signal generator (TG1304, Thurlby-Thandar, Huntingdon, UK). The diverging beam from the laser passes through a neutral density (ND) filter and is focused on a diffuser (ED1-S20, Thorlabs, Newton, MA, USA) by lens A (Bi-Convex, f = 20 mm). By selecting different levels of optical attenuation using the ND filter, different levels of optical signal power can be produced. The scattered light is collected by lens B (Bi-Convex, f = 20 mm) and uniformly illuminates over both photodetectors via a beam splitter. The reflected laser beam illuminates our 2D CMOS array while the transmitted laser beam passes through an aperture whose diameter is 2 mm and onto a reference photodiode (PDA520, Thorlabs, Newton, MA, USA) with a known transimpedance gain of 10^6^ V/A. The reference photodetector is used to calculate the amount of light falling on the 2D array.

The resultant output voltages from both detectors are then recorded using a PC based 16-bit ADC card (6034E, National Instruments, Austin, TX, USA) with a sampling duration of 1 s and stored on a PC. As the transimpedance gain of the reference photodetector is known, the reference photocurrent can be used to obtain the pixel AC and DC photocurrents and corresponding pixel AC transimpedance after appropriate scaling for detector area.

### 3.2. LED Phantom

**Figure 4.**
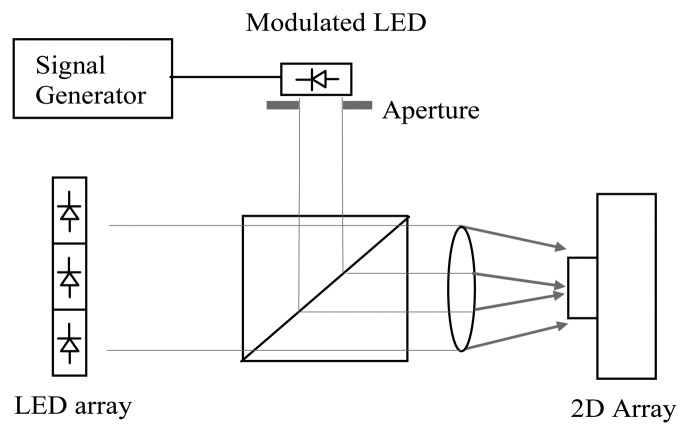
Experimental setup of the LED illumination.

The LED phantom provides predictable modulated signal to mimic a light level obtained from tissue. As shown in Figure 4, an array of eight light-emitting diodes (LED L53SRDG, Kingbright, Taipei, Taiwan) provides 640 nm constant light onto the sensor and a modulated LED connected to a signal generator (TG2000, Thurlby-Thandar, Huntingdon, UK) is used to produce modulated illumination at different frequencies and amplitudes. An aperture is used to control the size of the area with AC illumination over the sensor. By varying the aperture diameter, modulation depth and frequency, an inflammatory response which changes in flow and area can be simulated.

### 3.3. Rotating Diffuser

**Figure 5.**
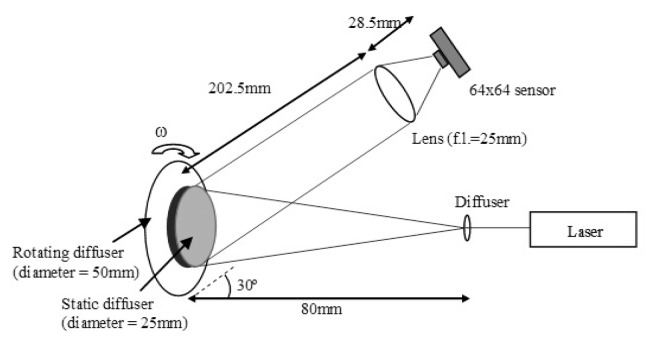
Experimental setup of the rotating diffuser.

The setup of the rotating diffuser test shown in Figure 5 allows different Doppler frequencies to be detected and analyzed by the system at different angular velocities (ω) of the diffuser. Laser light (λ = 780 nm, power = 40 mW) illuminates the surface of a static diffuser (diameter = 25 mm) placed in front of a rotating diffuser (diameter = 50 mm). The static diffuser is used to simulate a static layer of skin overlying moving red blood cells, from which the light scattered back is at optical frequency of f. Light scattered from the rotating diffuser is a frequency shifted signal (f + Δf) where Δf is the mean Doppler shifted frequency which is proportional to the angular velocity. The interference of these two backscattered signals at the sensor array allows the beat frequencies (Δf) to be detected.

### 3.4. In Vivo *Blood Flow Measurements*

**Figure 6.**
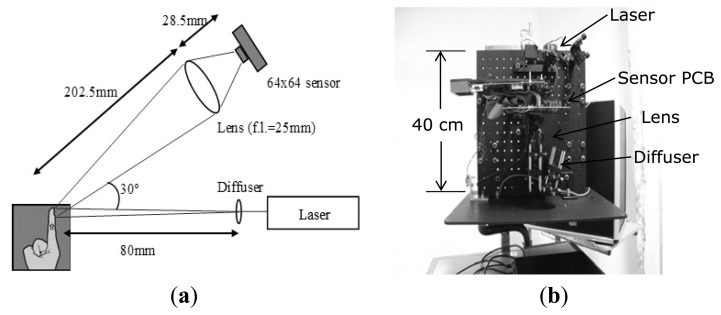
(**a**) Experimental setup of *in vivo* blood flow measurements; (**b**) Internal view of the device.

Figure 6a shows the setup of the *in vivo* blood flow experiments. Uniform illumination of 25 mm × 25 mm over the tissue is generated by a 40 mW diode laser (λ = 780 nm) and a diffuser (ED1-S20, Thorlabs). The illumination power density over the tissue is 64 μW/mm^2^. The backscattered light is then collected by an achromatic doublet lens (focal length = 25 mm) and imaged onto the sensor where there are ∼140 speckles at each pixel (speckle diameter = ∼2.1 μm). The speckle size is given by 2.44fλ(1+M)/D where f is the focal length, λ is the wavelength, M is the magnification and D is the clear aperture of the imaging lens [36]. Although this speckle size is not optimum in terms of spatial sampling and modulation depth, it allows sufficient light allow a 40 kHz sampling rate to be achieved. Problems associated with the low modulation depth are overcome by the on-chip processing. The whole optical system is embedded into a box, which connects to power supplies and a laptop for image display, as shown in Figure 6b. Studies in healthy participants have been approved by Southampton and South West Hampshire (UK) Research Ethics Committee (REC059/04/w). For calibration, measurements are made on a static tissue phantom and the baseline flow value (6 × 10^3^ a.u.) is subtracted from the measured flow.

## 4. Experimental Results

This section describes the results obtained with the experimental setups shown in Section 3.

### 4.1. Chip Characterization

**Figure 7.**
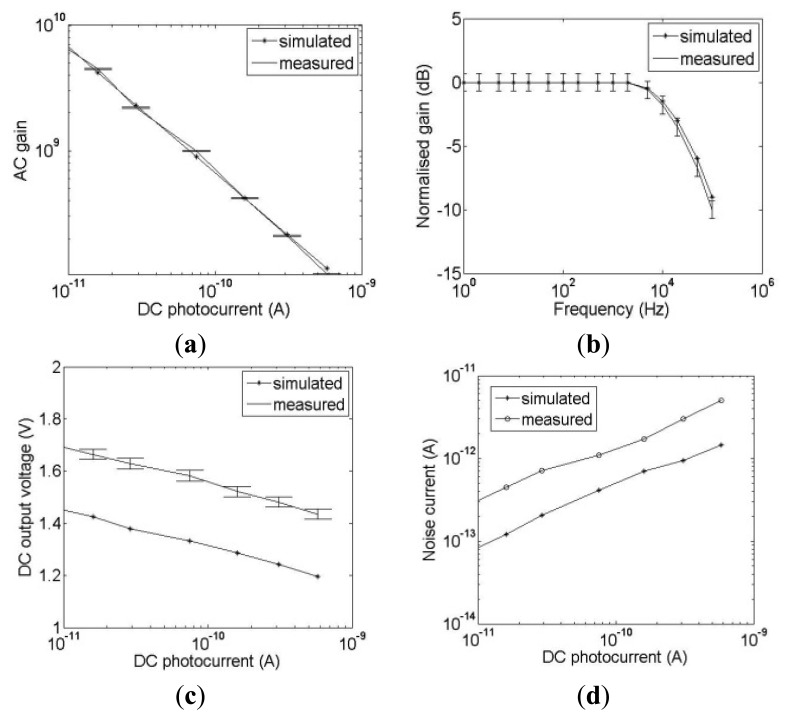
Measured and simulated results of the current to voltage converter characterization (**a**) AC gain (**b**) Frequency response at 150 pA DC photocurrent (**c**) DC response (**d**) Integrated noise current (rms).

Characterization of the sensor array was carried out using the system shown in Figure 3 in which the DC response of the current to voltage converter and AC responses of the current to voltage converter, HDA and GMC are characterized and compared with simulations. Figure 7 shows the characterization results for the current to voltage converter. Four main characteristics of AC gain, bandwidth, DC response and noise current are investigated. Figure 7a demonstrates that the AC gain is dependent on the DC photocurrent as expected from the logarithmic response described in Section 2. The error bars indicate the standard deviation of the AC gains for the 4096 pixels of the array. Typical simulation results obtained from a VLSI design tool, Cadence (Spectre, San Jose, CA, USA), are also plotted for comparison. Both measured and simulated AC gains are similar.

Figure 7b shows the measured and simulated frequency response of the current to voltage converter at 150 pA DC photocurrent with the gain normalized to 0 dB at DC. This DC photocurrent is selected because the cut-off frequency of the I/V converter is dependent on the DC photocurrent and an I_dc_ = 150 pA provides a cut off frequency that is close to the 20 kHz required for LDBF imaging. The −3 dB cut-off frequency from simulation is 22 kHz while that from measurement is 21 kHz. Figure 7c shows the measured DC output voltage of the current to voltage converter as the DC photocurrent is varied. The measured results are 200 mV higher than the simulated results which is due to the offset voltage of the buffers which drive the signal off chip. However this is acceptable because the DC output voltage is within the range of 1.4 V to 1.9 V which is within the input range of the HDA at the next stage. The HDA provides high amplification and a relatively flat response when the DC input voltage lies between 0.8 V to 2.2 V [33]. The standard deviation of 1.6% is shown by the error bars. Figure 7d shows that the measured noise is higher than that from simulations which is due to the amplitude fluctuations in the laser, power supply noise and other noise sources on the PCB. At 150 pA DC photocurrent, if the modulation depth is 1%, an SNR of 1.03 is obtained. If the modulation depth is 10% then the SNR = 10.3.

**Figure 8.**
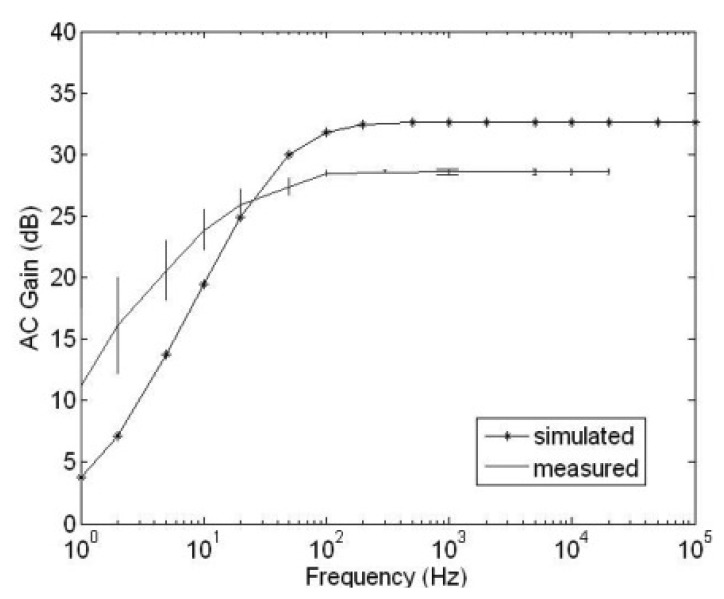
Measured and simulated frequency response of the HDA.

The frequency response of the HDA is characterized and shown in Figure 8. The lower cut-off frequency is 20 Hz while the simulation result is at 100 Hz which is likely to be due to the “inverted inverter” structure in the HDA which is not accurately modeled by the simulator.

**Figure 9.**
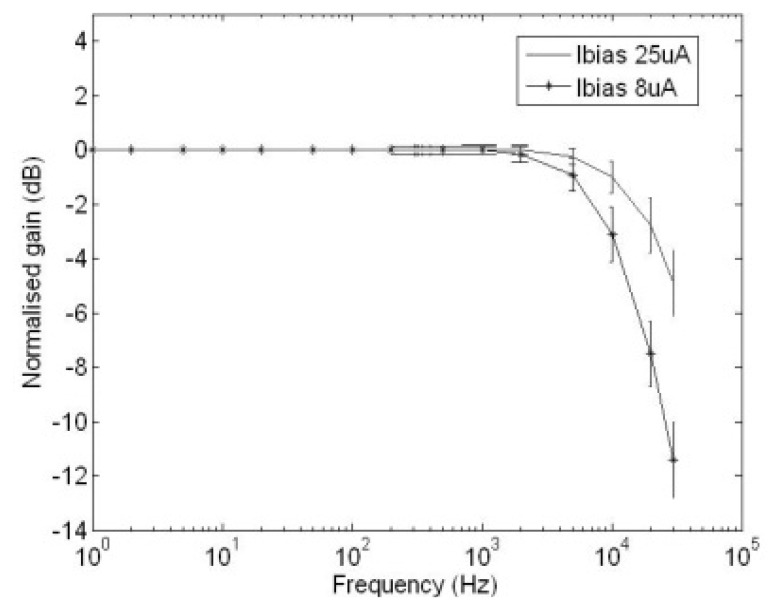
Measured frequency response of the GMC with the external bias currents of 8 μA and 25 μA.

Figure 9 illustrates the frequency responses of the GMC when the bias currents flowing through the external resistor are 8 μA and 25 μA respectively. It can be seen that in order to achieve a 20 kHz cut-off frequency from the GMC to avoid aliasing at a 40 kHz sampling frequency, the bias current of the GMC must be set at 8 μA.

### 4.2. LED Phantom

**Figure 10.**
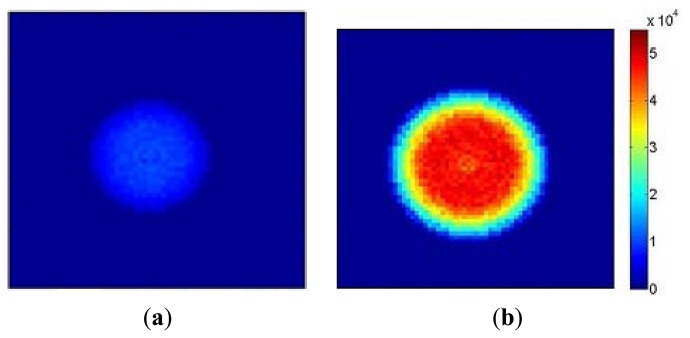
Simulated flow images (64 × 64 pixels) with (**a**) 5% modulation depth at 5 kHz and (**b**) 8% at 8 kHz.

Figure 10a shows a flow image obtained with an aperture diameter of 2 mm and 5% modulation depth at 5 kHz using the setup illustrated in Figure 4. As expected the high flow (1.3 × 10^4^ a.u.) at the central bright spot and the low flow (2 × 10^3^ a.u.) in the outer region can be observed. Figure 10b shows an image at an aperture diameter of 3 mm, 8% modulation depth and frequency of 8 kHz. The central bright spot expands and flow values also increase by a factor of approximately 4 (4.8 × 10^4^ a.u.) while the surrounding area maintains a low flow value. The results demonstrate that the sensor can discriminate between a modulated light signal in a relatively high DC background. As anticipated from Equation (1) the flow value increases linearly with modulation frequency and changes as the square of the modulation depth. The transition ring observed in Figure 10b was due to the edge effect of the aperture.

### 4.3. Rotating Diffuser

**Figure 11.**
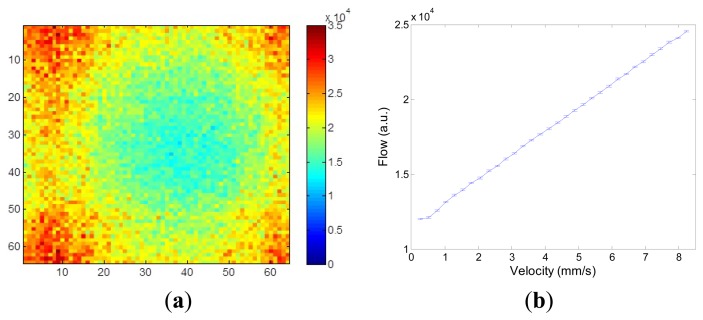
(**a**) Flow image of a rotating diffuser; (**b**) Flow averaged over all radial positions.

Figure 11a shows higher flow values towards the edge of the diffuser as the Doppler shift varies with radial position since the flow is velocity dependent as in Equation (1).

Figure 11b shows the radial profile of flow values averaged over all radial positions with the error bars denoting the standard deviation of the measurements at each radial position. As expected the averaged flow increases linearly as the velocity increases. At very low speed (<1 mm/s) an offset due to the noise floor of the system can be observed.

### 4.4. In Vivo *Blood Flow Measurements*

**Figure 12.**
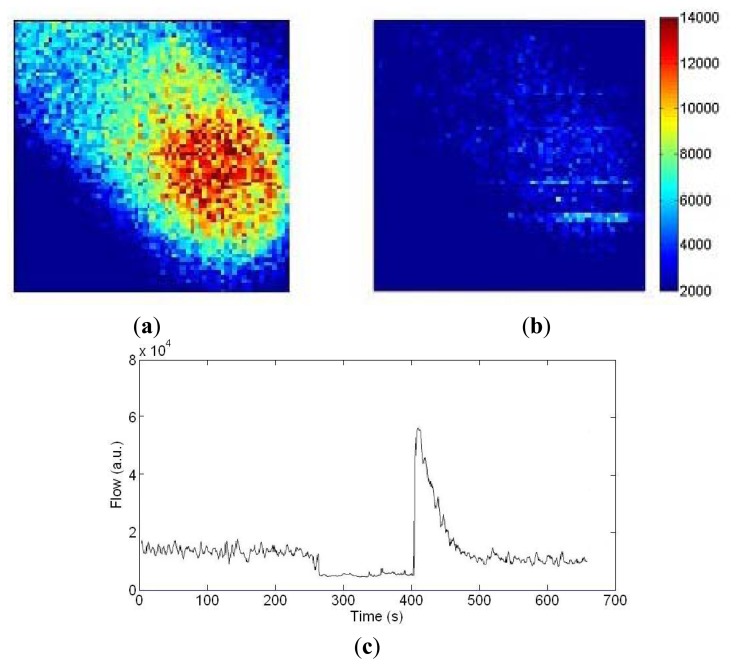
Blood flow measurements of a finger (the corresponding video clip is available online in the Supplementary Information for this paper). (**a**) Unoccluded; (**b**) Occluded; (**c**) Long-term blood flow trace of the center pixel.

Figure 12a,b shows blood flow images of a finger before and during arterial occlusion. The fingertip has higher blood flow (1.4 × 10^4^ a.u.) than the intermediate phalange (0.7 × 10^4^ a.u.). In Figure 12b the flow drops down to 0.3 × 10^4^ a.u. during occlusion. To demonstrate the long-term measurement capability, Figure 12c shows a trace of the flow values at the center pixel on the finger images with arterial occlusion at 180 mmHg for 2 min using a pressure cuff placed around the base of the finger. The transient increase in flow above resting flow during the post occlusive reactive hyperemia response can be observed.

**Figure 13.**
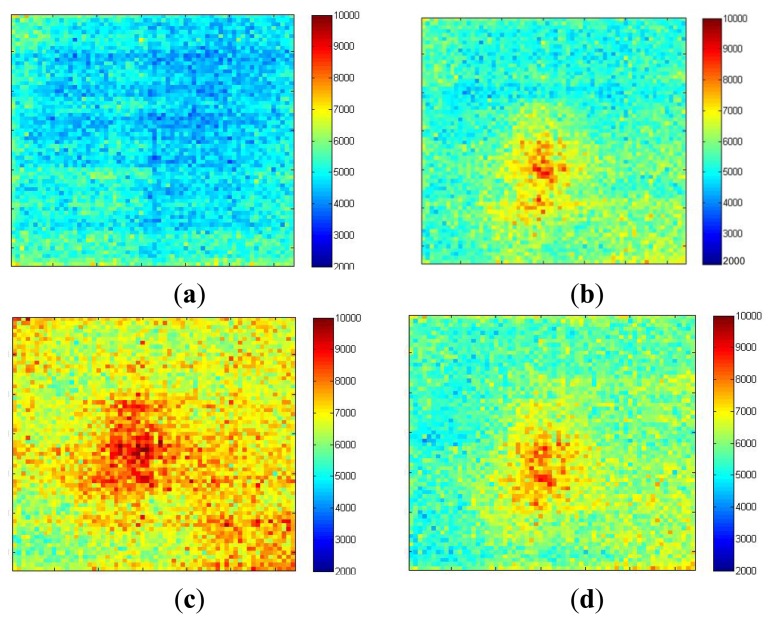
(**a**) Immediately after the histamine skin-prick; (**b**) 10 min after; (**c**) 20 min after; (**d**) 30 min after (field of view = 2.5 cm × 2.5 cm).

Imaging of an inflammatory response is carried out in order to demonstrate the sensor's ability to monitor gradually developing, sustained localized increases in blood flow. Ten mg/mL histamine was placed on the forearm, pricked gently with a sterile lancet (under ethical approval). The skin prick was performed as quickly as possible and imaging resumed immediately after. Figure 13a shows the blood flow image captured immediately after the histamine skin-prick. It has a relatively flat flow profile with an average of 4 × 10^3^ a.u. The horizontal stripe pattern illustrates the influence of blood pulsations during the data collection (discussed in Section 5). After 10 min (Figure 13b) a 0.5 cm × 0.5 cm circular area centered on the site of the skin prick developed with high flow (8 × 10^3^ a.u.). Figure 13c shows that after 20 min the inflammatory area expanded to 1 cm × 1 cm with increased flow of 9 × 10^3^ a.u. and the flow over the surrounding area also increased to 7 × 10^3^ a.u. After 30 minutes (Figure 13d) the size of the inflammatory area decreased to 0.6 cm × 0.6 cm with reduced flow of 8 × 10^3^ a.u.

## 5. Discussion and Conclusions

A 64 × 64 pixel fully integrated CMOS sensor for blood flow imaging is demonstrated. The sensor efficiently integrates analog and digital processing electronics on a single chip, which provides advantages over the existing blood flow imaging systems. The logarithmic response current to voltage converter provides natural normalization therefore the resource consuming divider is not required. The HDA circuit selectively amplifies the AC signal and hence significantly increases the AC/DC ratio far more space efficiently than implementing a conventional high pass filter on-chip. The pixel level anti-aliasing filter attenuates the noise above the signal bandwidth. Locally processing the laser Doppler signal means that large amounts of data do not need to be transferred off-chip, which is presently a data bottleneck. The sensor achieves 1 frame per second for 64 × 64 pixel blood flow images.

The analog subcircuits have been characterized individually and the results are consistent with simulations. The test results of the LED phantom and rotating diffuser demonstrate that the system can detect controlled modulated signals and show a linear relationship between the calculated flow and the Doppler shifted frequency. The changes of blood flow due to arterial occlusion can also be observed in the in vivo blood flow experiment. Finally the sensor images the blood flow changes due to the histamine skin-prick and shows the development of the inflammatory area with high flow. The images of tissue are influenced by blood pulsatile signals. This is because the sensor reads out the data by electronically scanning along the rows in each of the 32 × 32 pixel sub-arrays. As the overall frame rate is 1 fps (comparable to heart rate), there are positions during an image where blood volume is high (high signal in the rows) and where blood volume is low (low signal in the rows). This effect can be reduced by either averaging over frames or by employing a global shutter which will ensure that data at each pixel is obtained simultaneously.

Due to the advantages of AC amplification in the analog stage, the 10-bit ADC implemented here could be redesigned as an 8-bit version which would halve the area and increase the data conversion rate by a factor of 2. This allows ×8, 8 bit ADCs to be implemented in the same footprint as the ×4, 10 bit ADCs. Coupled with the factor of two improvement in conversion this would allow the sensor to achieve four frames per second. As the 64 × 64 array design is scalable to larger arrays, a 128 × 128 pixel array is planned consisting of four identical 64 × 64 sub-arrays. Each sub-array has 32 8-bit ADCs and a digital signal processor. The estimated silicon area is 12 mm × 13 mm and the sensor could achieve 16 frames per second.

## Figures and Tables

**Table 1.** Performance comparison of LDBF imaging systems.

**Name**	**Operational Mode**	**Image size (pixel)**	**No. of FFT points**	**Sampling frequency**	**Frame rate (per second)**	**Reference**
MoorLDI	Point scan	256 × 256	1024	40 kHz	0.004	[11]
MoorLDLS	Line scan	64 × 64	1024	40 kHz	0.25	[12]
TOPCAM	Full field	128 × 128	1024	27 kHz	0.2	[25]
Serov *et al.*	Full field	256 × 256	512	14 kHz	0.1	[24]
Leutenegger *et al.*	Full field	480 × 480	128	14.9 kHz	14.5	[26]
DOPCAM	Full field	64 × 64	1024	40 kHz	1	presented here
DOPCAM 2	Full field	128 × 128	1024	40 kHz	16	proposed
